# WKYMVm/FPR2 Alleviates Spinal Cord Injury by Attenuating the Inflammatory Response of Microglia

**DOI:** 10.1155/2022/4408099

**Published:** 2022-07-27

**Authors:** Wenwu Zhang, Jiewen Chen, Weimin Guo, Ganggang Kong, Le Wang, Xing Cheng, Xiaolin Zeng, Yong Wan, Xiang Li

**Affiliations:** ^1^Department of Spine Surgery, The First Affiliated Hospital of Sun Yat-sen University, Guangzhou, China; ^2^Guangdong Provincial Key Laboratory of Orthopaedics and Traumatology, Guangzhou, China

## Abstract

Spinal cord injury (SCI) is a common traumatic disease of the nervous system. The pathophysiological process of SCI includes primary injury and secondary injuries. An excessive inflammatory response leads to secondary tissue damage, which in turn exacerbates cellular and organ dysfunction. Due to the irreversibility of primary injury, current research on SCI mainly focuses on secondary injury, and the inflammatory response is considered the primary target. Thus, modulating the inflammatory response has been suggested as a new strategy for the treatment of SCI. In this study, microglial cell lines, primary microglia, and a rat SCI model were used, and we found that WKYMVm/FPR2 plays an anti-inflammatory role and reduces tissue damage after SCI by suppressing the extracellular signal-regulated kinases 1 and 2 (ERK1/2) and nuclear factor-*κ*B (NF-*κ*B) signaling pathways. FPR2 was activated by WKYMVm, suppressing the secretion of tumor necrosis factor-*α* (TNF-*α*), interleukin-6 (IL-6), and interleukin-1*β* (IL-1*β*) by inhibiting M1 microglial polarization. Moreover, FPR2 activation by WKYMVm could reduce structural disorders and neuronal loss in SCI rats. Overall, this study illustrated that the activation of FPR2 by WKYMVm repressed M1 microglial polarization by suppressing the ERK1/2 and NF-*κ*B signaling pathways to alleviate tissue damage and locomotor decline after SCI. These findings provide further insight into SCI and help identify novel treatment strategies.

## 1. Introduction

Spinal cord injury (SCI), which is usually caused by serious trauma such as falls and traffic accidents, is one of the most serious injuries of the central nervous system (CNS) and has a high rate of disability and serious complications [1, 2]. Primary and secondary injuries are consecutive and overlapping pathological processes of SCI [3]. Primary SCI is caused by direct mechanical impact and is characterized by bruising, compression, kinking, and stretching. In SCI, primary injury is considered irreversible. The pathogenesis of secondary SCI is complex and is continuously regulated at the intracellular and extracellular molecular levels; major events include lipid peroxidation, mitochondrial dysfunction, oxidative stress, inflammatory response, nerve cell apoptosis, and glutamate receptor overactivation [3–5]. Through a series of molecular cascade reactions, the degree of tissue damage is further exacerbated, and the scope of SCI is expanded. The role of inflammation in the progression of SCI cannot be ignored [6, 7]. After SCI, the expression of inflammatory cytokines and chemokines in the injured site is upregulated, which not only induces the death of neuronal cells and immune cell infiltration but also promotes the inflammatory cascade and worsens the inflammatory microenvironment [8, 9]. Therefore, effectively reducing the inflammatory response during secondary injury has become a new entry focus in the study of SCI treatment strategies.

Microglia, which are innate immune cells in the CNS, regulate innate and adaptive immune responses in a series of pathophysiological processes [10, 11]. Activated microglia can modulate the response to pathogens and perform immune cell functions by clearing cell debris through phagocytosis [12, 13]. In addition, microglia are involved in both beneficial and harmful responses during SCI [14]. This paradoxical role is associated with activated and polarized microglial subtypes. The classic concept of microglial polarization holds that M1 polarization represents the proinflammatory phenotype, while M2 polarization represents the anti-inflammatory phenotype [15, 16]. During the development of SCI, M1-polarized microglia produce a large number of proinflammatory cytokines, which are harmful to both damaged and healthy neurons [17, 18] and lead to secondary injury [19]. In contrast, M2-polarized microglia can upregulate anti-inflammatory mediator production and are involved in limiting inflammation and restoring homeostasis [20, 21]. Therefore, inhibiting M1 polarization and promoting M2 polarization in microglia are methods worth exploring in the study of SCI.

It has been reported that there are many signaling pathways involved in regulating the inflammatory response of microglia. Nuclear factor-*κ*B (NF-*κ*B), a clear inflammatory regulatory pathway, has been reported to promote the expression and release of proinflammatory cytokines, many of which are upregulated in SCI [22]. Nicolas and colleagues reported that NF-*κ*B signaling was closely associated with the proinflammatory phenotype and M1 microglial polarization [23]. Mitogen-activated protein kinases (MAPKs) are considered important signaling pathways involved in cellular stress and the inflammatory response [24–27]. The increased phosphorylation of p38MAPK and extracellular signal-regulated kinases 1 and 2 (ERK1/2) in microglia under pathological conditions is responsible for the increased expression of proinflammatory cytokines such as tumor necrosis factor-*α* (TNF-*α*), interleukin-6 (IL-6), and interleukin-1*β* (IL-1*β*) [25–28]. These studies suggest that NF-*κ*B and MAPKs are the classic signaling pathways associated with the inflammatory response.

Formyl peptide receptors (FPRs), which include the isomers FPR1, FPR2/ALX, and FPR3 [29], belong to the G-protein-coupled receptor family [30, 31]. FPRs are crucial regulatory targets due to their involvement in a number of inflammatory diseases [32] and play important roles in host defense and regulation of the inflammatory response. Vital SA and colleagues demonstrated that FPRs are expressed in many structures of the brain and spinal cord [33]. Recently, many studies have shown that FPR2 is a key member of the FPR family and has a significant anti-inflammatory effect [29, 34]. Loss of the FPR2 receptor has been shown to contribute to disease progression [33]. Therefore, FPR2 may be an important target for intervening in secondary inflammatory processes and may be used to develop a novel therapeutic strategy for treating SCI.

WKYMVm is a synthetic peptide that was identified from peptide libraries, is a selective FPR2 agonist [35, 36], and exerts an immunoregulatory effect [37]. The anti-inflammatory properties of this peptide are gradually being discovered, and it has been shown to significantly inhibit the release of inflammatory cytokines such as TNF-*α* and IL-1*β* [38]. Moreover, compared with other FPR2 agonists, this peptide has the advantages of better affinity and low immunogenicity [37]. Therefore, WKYMVm/FPR2 may effectively interfere with the inflammatory response in SCI, and the application of WKYMVm/FPR2 in SCI has improved prospects. However, there have been few reports on the role of WKYMVm and FPR2 in microglia. In particular, their roles and underlying mechanisms in SCI are not clear.

Based on this logic, we believe that WKYMVm may play an inhibitory role in the inflammatory response of microglia through FPR2 and may become an agent for regulating inflammation and treating SCI. To test this hypothesis, we investigated the role of WKYMVm and FPR2 in the treatment of SCI through microglial cell culture in vitro and SCI experiments in rats. In addition, we attempted to elucidate the mechanism by which WKYMVm/FPR2 regulates microglia-associated inflammation. Our data suggested that WKYMVm/FPR2 has significant anti-inflammatory effects in vivo and in vitro and reduces tissue damage in rats with SCI. In addition, the mechanism of these effects involves WKYMVm mediation of FPR2 activation, which suppresses the ERK1/2 and NF-*κ*B signaling pathways. These results suggest that WKYMVm negatively regulates the inflammatory response during SCI in rats and that WKYMVm/FPR2 may be a novel therapeutic target for SCI, which deserves further research and development.

## 2. Materials and Methods

### 2.1. Reagents and Antibodies

WKYMVm was acquired from Gibco Technology (Montclair, CA, USA), and its purity was ≥98%. The lipopolysaccharide (LPS) was purchased from Sigma-Aldrich (St Louis, MO, USA). The primary antibodies for FPR2, iNOS, and Iba-1 were provided by Novus Biologicals (Novusbio, CO, USA). The p-p38, p38, p-ERK, ERK, p-NF-*κ*B p65, NF-*κ*B p65, I*κ*B*α*, and *β*-tubulin primary antibodies were purchased from Affinity (Affinity Biosciences, Jiangsu, China). 4′-6-Diamidino-2-phenylindole (DAPI) was purchased from Phygene (Fuzhou, China). Alexa-Fluor-488- and Alexa-Fluor-594-tagged secondary antibodies were obtained from Abcam (Boston, MA, USA). The reagents for cell culture were purchased from Gibco Technology (Grand Island, NY, USA). The enzyme-linked immunosorbent assay (ELISA) kits of rat TNF-*α*, IL-6, and IL-1*β* were obtained from NEOBIOSCIENCE (Shenzhen, China). Evo M-MLV RT Premix for qPCR was obtained from Accurate Biotechnology Co., Ltd (Changsha, China). NovoStart® SYBR qPCR SuperMix Plus was provided by Novoprotein (Shanghai, China).

### 2.2. Pharmaceutical Inhibitors

Specific inhibitors used in cell experiments are as follows: p38-specific inhibitor (SB203580, Gibco, USA), ERK-specific inhibitor (U0126, Gibco, USA), and NF-*κ*B p65-specific inhibitor (BAY11-7082, Gibco, USA). The cells were pretreated with these pharmaceutical inhibitors for 1 hour, followed by other treatments.

### 2.3. Primary Microglia Isolation and Culture

Primary microglia were prepared as follows [39]. Sprague-Dawley neonatal rats on the first day of birth were purchased from the animal Laboratory of Sun Yat-sen University. The rats were decapitated, and their skulls were cut with ophthalmic scissors to remove their brains. The cerebral cortex were isolated and chopped with a scalpel, then treated with 0.25% Trypsin-EDTA (Gibco, USA) at 37°C for 10 min. Digestion was suspended with complete culture medium composed of 90% DMEM/F12, 10% fetal bovine serum (FBS), penicillin (100 units/ml), and streptomycin (100 mg/ml). Mixture was filtered using a 100 mm cell strainer. Cells were then seeded in a flask and cultured in a 5% CO2 incubator at 37°C. After 14 days of primary cultivation, microglial cells were collected by shaking and replating.

Highly aggressive proliferating immortalized (HAPI) microglia cells, which are a rat microglial cell line, were obtained from BLUEFCELL (BFB, Shanghai, China). HAPI microglial cells were cultured in a complete culture medium composed of 90% DMEM, 10% FBS, penicillin (100 units/ml), and streptomycin (100 mg/ml) in an incubator set at a constant temperature of 37°C with 5% CO2. HAPI microglial cells and primary microglia were treated with or without different concentrations of WKYMVm for 2 hours, and then LPS was added into the culture media at specific concentrations for different periods.

### 2.4. Cytotoxicity Assay

WKYMVm cytotoxicity to HAPI microglial cells and primary microglia was examined using a CCK-8 kit. HAPI microglial cells and primary microglia were seeded in 96-well plates at a density of 5 × 103 per well and were treated with a range of WKYMVm concentrations for 12 hours, 24 hours, or 48 hours. Next, the CCK-8 kit solution (10 *μ*l/well) was added to the 96-well plates, which were then incubated in the dark for 1 hour. Absorbance was detected at 450 nm and recorded by a microplate reader.

### 2.5. SCI Animal Model and Treatment

Adult female Sprague-Dawley rats (200-220 g) were obtained from the Beijing Charles River Laboratory Animal Technology Co., Ltd (Beijing, China). The animals were rested for 7 days to adapt to the environment before surgical procedures. Spinal cord injury protocols in rats were described previously [40]. Briefly, rats were anesthetized with 1% pentobarbital sodium (40-45 mg/kg) to cause minimum pain. The operator performed a laminectomy located at the T9 vertebral section and removed the skin and muscles around the spinous processes to expose the vertebral column. The spinal cord was explicitly exposed and clamped using a vascular clip (60 g force, FST, China) for 60 seconds to establish a moderate crushing injury model. For the sham group, T9 laminectomy was performed, and the spinal cord was exposed for 60 seconds without compression injury. After surgery, rats from the SCI+WKYMVm group were intraperitoneally injected with WKYMVm (dissolved in 0.9% sodium chloride) at a dose of 4 mg/kg body weight. In contrast, the rats from the sham group and SCI group were intraperitoneally injected with 0.9% sodium chloride at the same volume. Injections were performed intraperitoneally altogether 3 times with 24 hours intervals between applications. The rats were housed for 12 h of light/dark cycle under standard temperature conditions, with feeding and drinking water. After surgery, the bladder was emptied twice a day until bladder function was restored.

### 2.6. Western Blot Analysis

Spinal cord tissue was collected at a specific time after operation according to experimental requirements. Briefly, the spinal cord tissue and microglial cells were lysed in RIPA buffer, and then the protein concentrations were measured with a bicinchoninic acid disodium (BCA) assay. Total proteins were subjected to sodium dodecyl sulfate polyacrylamide gel electrophoresis (SDS-PAGE) and then transferred by electroblotting to polyvinylidene difluoride (PVDF) membranes. After blocking with 5% nonfat dry milk for 1 hour and washing with TBST (Tris-HCl-based buffer with Tween 20) buffer for 30 minutes, specific primary antibodies against the proteins were incubated with the membranes in the specified dilutions overnight at 4°C. Then, the membranes were incubated with horseradish peroxidase- (HRP-) conjugated secondary antibodies for 1 hour at room temperature and washed three times with TBST. Immunolabeling was detected by an ECL reagent (Thermo Fisher Scientific). The resultant signals were detected using the ImageQuant Las4000mini (GE, Japan), and quantification of protein density was done by the ImageJ software. *β*-Tubulin served as an internal control.

### 2.7. RNA Isolation and Real-Time PCR

After treatment, total RNAs were extracted from the HAPI microglial cells, primary microglia, and homogenates of spinal cord tissue using the TRIzol® reagent (Invitrogen, CA, USA) according to the manufacturer's instructions. RNA concentrations were measured by the spectrophotometer. For PCR amplification, 10 *μ*l of reaction volume was used, including 5 *μ*l of 2× NovoStart® SYBR qPCR SuperMix Plus (Shanghai, China), 0.2 mmol/L of each primer, 2 *μ*l of 2-fold diluted cDNA, and sterile distilled water. The reaction and detection were conducted in a light cycler (Roche, Switzerland). Primer sequences used are listed in Table 1. The cycle threshold (Ct) values were collected and normalized to the level of the housekeeping gene GAPDH.

### 2.8. Immunohistochemistry (IHC)

The sections of rat spinal cord tissues (T9 transverse) were deparaffinized, rehydrated, and performed heat-mediated antigen retrieval with sodium citrate at 95°C for 15 min. The sections were incubated with 3% H_2_O_2_ for 15 min and washed three times in phosphate-buffered saline (PBS). Then the sections were incubated with 0.25% Triton X-100 for 10 min. After washing three times in PBS, the sections were blocked with 10% goat serum and incubated overnight with anti-iNOS primary antibody (1 : 50) at 4°C. The next day, the slices were cleaned to remove the primary antibody and then incubated with HRP-conjugated secondary antibody at room temperature for 60 min, and 3,3′-diaminobenzidine was used to generate signals. All images were observed using a microscope (Leica DMI4000B, Wetzlar, Germany).

### 2.9. Immunofluorescence and Histologic Analyses

Immunofluorescence was performed on cells or tissues. Primary microglia were fixed with 4% PFA for 20 minutes. Rat spinal cord tissues were deparaffinized and rehydrated. The tissue or cell slices were permeabilized with 0.25% Triton X-100 for 20 minutes and blocked with 10% Normal Donkey Serum for 30 minutes. Then, they were incubated with the following primary antibodies overnight at 4°C: anti-FPR2 (1 : 50), anti-iNOS (1 : 50), and anti-Ib*α*-1 (1 : 300). The next day, after washing three times with PBS, the primary antibodies were probed with the following secondary antibodies for 1 hour at room temperature: Alexa-Fluor 488 donkey anti-rabbit and Alexa-Fluor 594 donkey anti-goat. Finally, the slices were labeled with DAPI after washing three times with PBS. All images were observed using an Olympus BX63 microscope (Olympus, Japan).

To evaluate the extent of the injury and neuronal loss, a histopathological examination was performed with haematoxylin and eosin (H&E) and Nissl staining as per the manufacturer's instructions. A bright-field microscope (Olympus, Tokyo, Japan) was used to observe and obtain images.

### 2.10. Enzyme-Linked Immunosorbent Assay (ELISA)

Supernatants were collected from cell culture and tissue lysis and subsequently assayed for cytokine production. The cytokines TNF-*α*, IL-6, and IL-1*β* were assessed using NeoBiosence ELISA kits (Shenzhen, China) following the manufacturer's protocol. The data were acquired by using a Multiskan Sunrise microplate reader (TECAN, Austria).

### 2.11. Locomotion Function Assessment

Locomotion function was assessed by using Basso-Beattie-Bresnahan (BBB) scores and footprint analysis after SCI. The range of BBB scores which includes the evaluation of gait, joint motion, limb coordination, and torso stability was from 0 (no limb movement or weight support) to 21 (normal locomotion). Rats were allowed to move freely in an open experimental field. For footprint analysis, rat posterior and anterior limbs were dipped with red and blue dyes, respectively. Motor functional evaluation of rats was conducted by two experienced observers blinded to the experimental conditions.

### 2.12. Statistical Analysis

All data were expressed as a means ± SEM format for at least three independent experiments. Statistical significance was analyzed with a one-way analysis of variance (ANOVA) using GraphPad Prism 8 (La Jolla, CA, USA) for Windows. A value of *p* < 0.05 indicated statistical significance.

## 3. Results

### 3.1. WKYMVm Had No Effect on Microglial Proliferation or Viability

**Figure 1:**
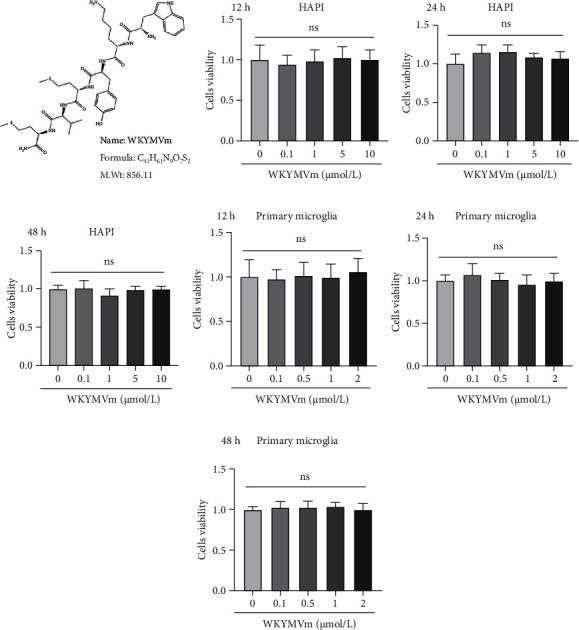
WKYMVm had no effect on microglial proliferation or viability. (a) The molecular structure of WKYMVm. Cytotoxic effects of WKYMVm on HAPI microglia and primary microglia were examined by the CCK-8 assay. (b–d) HAPI microglia were incubated with increasing concentrations (0, 0.1, 1, 5, and 10 *μ*mol/l) of WKYMVm for 12 h, 24 h, and 48 h at 37°C. (e–g) Primary microglia were incubated with increasing concentrations (0, 0.1, 0.5, 1, and 2 *μ*mol/l) of WKYMVm for 12 h, 24 h, and 48 h at 37°C. M.Wt: molecular weight.

The molecular structure of WKYMVm is shown in Figure 1(a). To examine the effect of WKYMVm on microglial proliferation and viability, a CCK-8 kit was used. Microglial cells (including HAPI microglia and primary microglia) were incubated with a range of WKYMVm concentrations for 12, 24, and 48 hours. As shown in Figures 1(b)–1(d), WKYMVm at concentrations ≤ 10 *μ*mol/l did not affect the proliferation or viability of HAPI microglia compared with those in the control group. Similarly, WKYMVm at concentrations ≤ 2 *μ*mol/l did not affect the proliferation or viability of primary microglia (Figures 1(e)–1(g)). These results showed that 0~10 *μ*mol/l WKYMVm stimulated HAPI microglial cells and 0~2 *μ*mol/l WKYMVm stimulated primary microglia without inducing cytotoxicity.

### 3.2. WKYMVm Promoted the Expression of FPR2 in Microglial Cells

**Figure 2:**
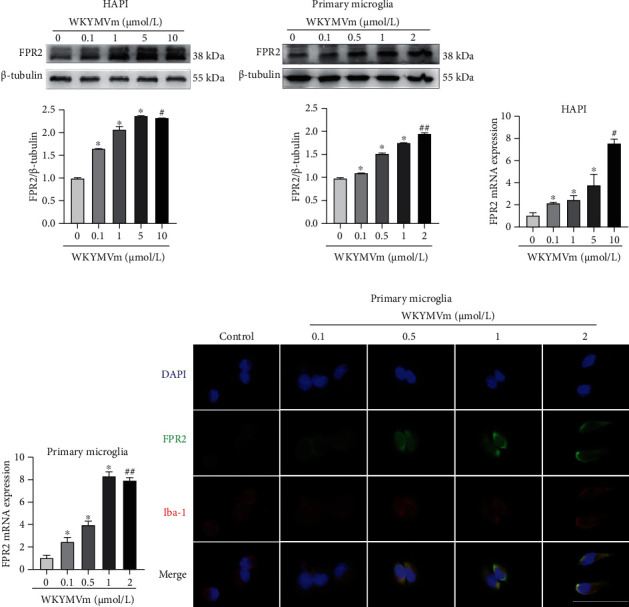
WKYMVm promoted the expression of FPR2 in microglial cells. (a, b) Western blots and quantitative data for FPR2 expression in HAPI microglia and primary microglia stimulated by different concentrations of WKYMVm. (c, d) FPR2 mRNA levels in HAPI microglia and primary microglia are stimulated by different concentrations of WKYMVm. (e) Immunofluorescence staining for FPR2 in primary microglia stimulated by WKYMVm with concentrations from 0 to 2 *μ*mol/l. scale bar: 50 *μ*m. ^∗^*p* < 0.05 vs. the control group; ^#^*p* < 0.05 vs. the 5 *μ*mol/l WKYMVm group; ^##^*p* < 0.05 vs. the 1 *μ*mol/l WKYMVm group.

To investigate the effects of WKYMVm on FPR2 expression in microglial cells, we evaluated the expression of FPR2 in HAPI microglia and primary microglia in response to different concentrations of WKYMVm. Western blot analysis showed that the expression of FPR2 in both microglial cells increased with increasing WKYMVm concentrations (Figures 2(a) and 2(b)). Further real-time PCR verified this trend (Figures 2(c) and 2(d)). In addition, we performed immunofluorescence costaining of FPR2 and Iba-1 (a classical marker of microglia) in primary microglia. Consistent with the Western blot analysis and real-time PCR results, FPR2 fluorescence intensity was increased with increasing WKYMVm concentrations (Figure 2(e)). These results suggest that WKYMVm promotes the expression of FPR2 in microglial cells in a concentration-dependent manner. In this study, we chose 5 *μ*mol/l and 1 *μ*mol/l as the treatment concentrations for HAPI microglia and primary microglia, respectively.

### 3.3. WKYMVm Inhibited Inflammatory Cytokine Production in Microglial Cells

**Figure 3:**
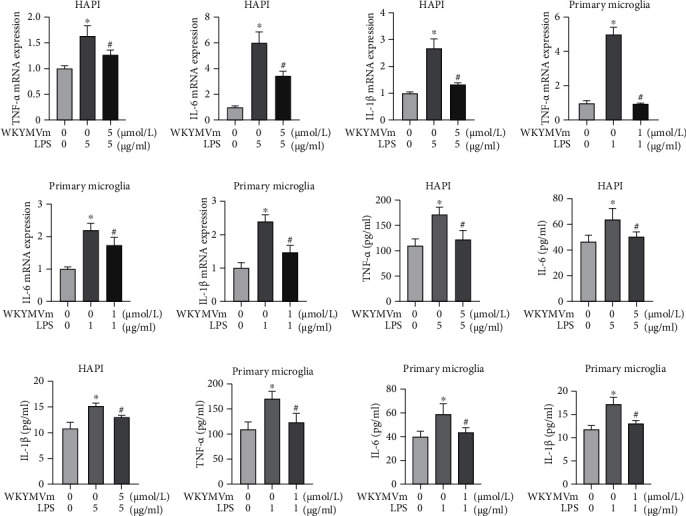
WKYMVm inhibited inflammatory cytokine production in microglial cells. (a–c) Expression levels of TNF-*α*, IL-6, and IL-1*β* in HAPI microglia from each group were detected by RT-PCR assay. (d–f) mRNA levels of TNF-*α*, IL-6, and IL-1*β* in primary microglia from each group. (g–i) The protein content of TNF-*α*, IL-6, and IL-1*β* in the supernatant of the HAPI microglia culture medium was detected by ELISA. (j–l) The protein level of TNF-*α*, IL-6, and IL-1*β* in the supernatant of the primary microglia culture medium. ^∗^*p* < 0.05 vs. the control group; ^#^*p* < 0.05 vs. the LPS group.

The secretion of proinflammatory cytokines indicates the presence of an inflammatory response. To validate the anti-inflammatory effect of WKYMVm, we examined the effects of WKYMVm on the production of proinflammatory cytokines (TNF-*α*, IL-6, and IL-1*β*) in LPS-stimulated microglial cells. Stimulated with LPS, HAPI microglia and primary microglia displayed higher mRNA levels of TNF-*α*, IL-6, and IL-1*β* than the control group. Notably, WKYMVm significantly decreased the gene levels of these cytokines in microglial cells (Figures 3(a)–3(f)). ELISA also showed that TNF-*α*, IL-6, and IL-1*β* production was significantly increased in the supernatants of LPS-stimulated microglial cells, whereas WKYMVm downregulated the levels of these cytokines in the supernatants of HAPI microglia (Figures 3(g)–3(i)) and primary microglia (Figures 3(j)–3(l)). Thus, our results indicated that WKYMVm had an inhibitory effect on the inflammatory response in microglial cells.

### 3.4. WKYMVm Inhibited M1 Microglial Polarization

**Figure 4:**
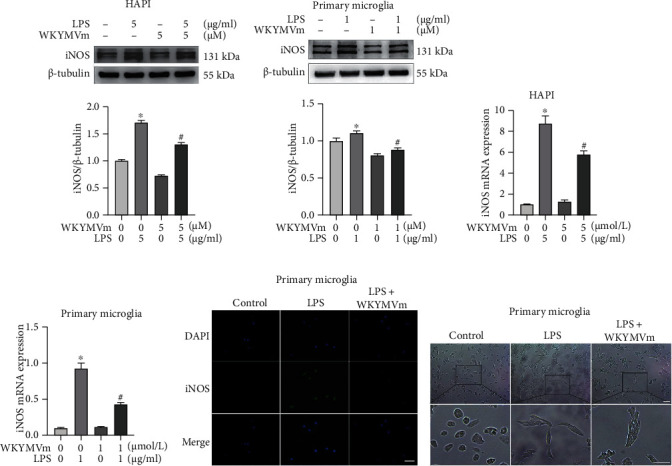
WKYMVm inhibited M1 microglial polarization. iNOS is one of the markers of M1 microglial cells. (a, b) Western blots and quantitative data for iNOS protein in HAPI microglia and primary microglia from different groups. (c, d) The expression of iNOS mRNA in HAPI microglia and primary microglia from each group was detected by RT-PCR assay. (e) Immunofluorescence staining for iNOS in primary microglia from different groups. Scale bar: 50 *μ*m. (f) Morphological results of primary microglia treated with LPS or WKYMVm. Scale bar: 100 *μ*m. ^∗^*p* < 0.05 vs. the control group; ^#^*p* < 0.05 vs. the LPS group.

M1-polarized microglia, which represent proinflammatory subtypes, can lead to an increase in proinflammatory cytokines [41]. iNOS is considered one of the markers of M1 microglial polarization [42]. To explore the effect of WKYMVm on microglial cell polarization, we examined the iNOS protein and mRNA levels in LPS-stimulated microglial cells with or without pretreatment with WKYMVm. Western blot analysis showed that the iNOS protein level was elevated by LPS stimulation compared with the control group, and WKYMVm significantly inhibited the production of iNOS in LPS-stimulated HAPI microglia (Figure 4(a)) and primary microglia (Figure 4(b)). Consistent with changes in protein levels, iNOS gene levels were markedly increased in LPS-stimulated HAPI microglia and primary microglia, and pretreatment with WKYMVm significantly reduced iNOS mRNA levels (Figures 4(c) and 4(d)). In addition, WKYMVm reversed the increasing fluorescence intensity of iNOS in LPS-induced primary microglia (Figure 4(e)). Moreover, primary microglia turned into amoeba-like shapes after stimulation with LPS, which indicated the morphological manifestations of the M1 subtype, and this effect was partially reversed by treatment with WKYMVm (Figure 4(f)). Taken together, these results suggested that LPS induced the M1 polarization of microglial cells, and WKYMVm reversed this effect.

### 3.5. WKYMVm Inhibited the ERK1/2 and NF-*κ*B p65 Signaling Pathways and Affected the p38 Signaling Pathway in Microglial Cells

**Figure 5:**
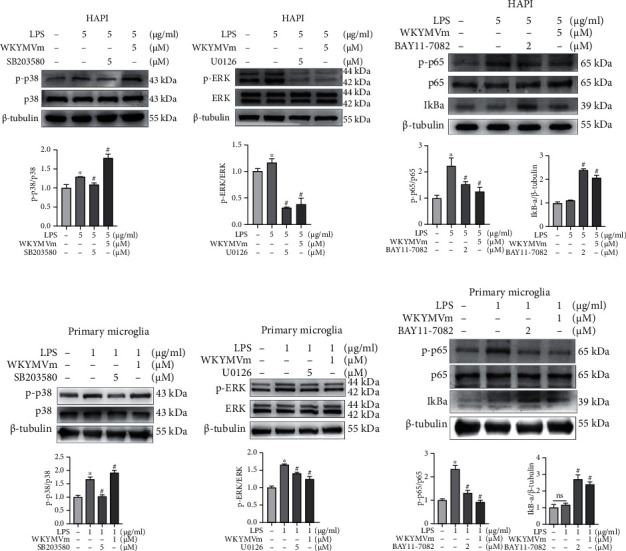
WKYMVm inhibited the ERK1/2 and NF-*κ*B p65 signaling pathways and affected the p38 signaling pathway in microglial cells. (a–c) Western blot analyses and quantitative data. The impact of FPR2 activation by WKYMVm on phosphorylation of p38, ERK1/2, NF-*κ*B p65, and I*κ*B*α* protein level in LPS-treated HAPI microglia with or without pretreatment of WKYMVm. (d–f) Western blot analyses and quantitative data for p-p38, p-ERK1/2, p-NF-*κ*B p65, and I*κ*B*α* from each group in primary microglia. SB203580: p38-specific inhibitor; U0126: ERK-specific inhibitor; BAY11-7082: NF-*κ*B p65-specific inhibitor. ^∗^*p* < 0.05 vs. the control group; ^#^*p* < 0.05 vs. the LPS group.

MAPKs, including p38 and ERK1/2, play an important role in inflammatory regulation; therefore, we examined whether the activation of WKYMVm/FPR2 affected the MAPK signaling pathway in LPS-induced microglial cells. Western blot analysis was used to determine whether WKYMVm could inhibit LPS-induced phosphorylation of P38 and ERK1/2 in HAPI microglia and primary microglia. The protein expressions of p-p38, p38, p-ERK1/2, ERK1/2, and *β*-tubulin in the control group, LPS group, LPS+SB203580 group, LPS+U0126 group, and LPS+WKYMVm group were analyzed. The findings showed that LPS surely enhanced the phosphorylation of p38, and this effect was inhibited by SB203580. However, p-p38 was enhanced by WKYMVm in HAPI microglia and primary microglia (Figures 5(a) and 5(d)). Similarly, LPS markedly increased the level of p-ERK1/2, but this trend was effectively inhibited by U0126 and WKYMVm (Figures 5(b) and 5(e)).

The NF-*κ*B p65 signaling pathway is one of the most important regulators of microglial polarization, and its activation is accompanied by the phosphorylation of p65 and degradation of the NF-*κ*B inhibitor I*κ*B*α* [43, 44]. To determine the anti-inflammatory mechanism of activated WKYMVm/FPR2, we examined the levels of the key NF-*κ*B signaling pathway mediators p65 and I*κ*B*α* in LPS-stimulated HAPI microglia and primary microglia. The protein expression of p-NF-*κ*B p65, NF-*κ*B p65, I*κ*B*α*, and *β*-tubulin was analyzed by western blotting in the control group, LPS group, LPS+BAY11-7082 group, and LPS+WKYMVm group. Compared with that in the control group, the phosphorylation of NF-*κ*B p65 was significantly increased in the LPS group, while BAY11-7082 and WKYMVm effectively inhibited this effect (Figures 5(c) and 5(f)). As expected, LPS-induced total I*κ*B*α* protein degradation was markedly inhibited by BAY11-7082 and WKYMVm (Figures 5(c) and 5(f)). Therefore, ERK1/2 and NF-*κ*B p65 may be crucial signaling pathways involved in the inflammatory process in microglial cells, and the anti-inflammatory effect of FPR2 activation by WKYMVm may be related to these pathways.

### 3.6. WKYMVm Inhibited the ERK1/2 and NF-*κ*B p65 Signaling Pathways and Affected the p38 Signaling Pathway after SCI

**Figure 6:**
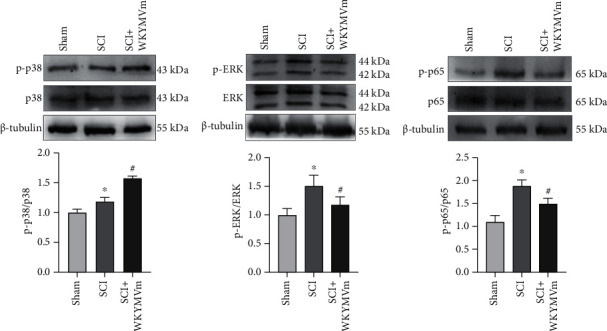
WKYMVm inhibited the ERK1/2 and NF-*κ*B p65 signaling pathways and affected the p38 signaling pathway after SCI. (a–c) Western blots and quantitative data for p-p38, p-ERK1/2, and p-p65 protein in tissue at 72 h after SCI. ^∗^*p* < 0.05 vs. the sham group; ^#^*p* < 0.05 vs. the SCI group.

Based on the effects of WKYMVm on the p38, ERK1/2, and NF-*κ*B p65 signaling pathways in vitro, we further verified the results in vivo. Western blot assays were used to assess whether WKYMVm could inhibit p-p38, p-ERK1/2, and p-p65 protein expression in SCI. The results showed that the phosphorylation of p38 was increased in the SCI group, while WKYMVm enhanced it (Figure 6(a)). However, ERK1/2 and p65 phosphorylation were significantly increased in SCI, and this effect was markedly inhibited by WKYMVm (Figures 6(b) and 6(c)). Our data showed that SCI significantly enhanced p38, ERK1/2, and NF-*κ*B p65 phosphorylation, and WKYMVm reduced the phosphorylation of ERK1/2 and NF-*κ*B p65 but not p38.

### 3.7. WKYMVm Attenuated M1 Polarization after SCI

**Figure 7:**
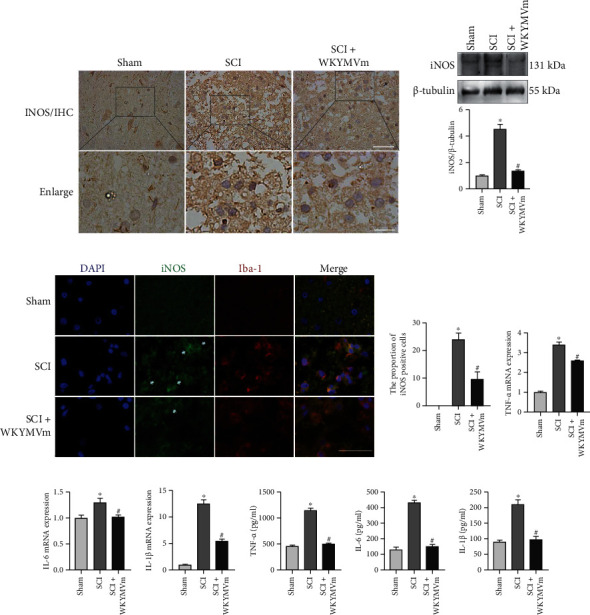
WKYMVm attenuated M1 polarization and improved the inflammatory microenvironment after SCI. (a) Immunohistochemical displayed the iNOS signal in sections from the tissue at 72 h after SCI. Scale bars: 50 *μ*m (INOS/IHC); 20 *μ*m (enlarge). (b) Western blots and quantitative data for iNOS protein expression in sections from the tissue at 72 h after SCI. (c) Double immunofluorescence staining and quantitative data for iNOS- (green) and Iba-1- (red) positive microglia of sections from the tissue at 72 h after SCI. White arrows mark positive cells. Scale bar: 50 *μ*m. (d–f) The levels of TNF-*α*, IL-6, and IL-1*β* mRNA in tissue from SCI rats treated with or without WKYMVm were measured by RT-PCR at 72 h. (g–i) Quantification analysis of the production of TNF-*α*, IL-6, and IL-1*β* in the spinal cord after SCI by ELISA. ^∗^*p* < 0.05 vs. the sham group; ^#^*p* < 0.05 vs. the SCI group.

Based on the inhibitory effect of WKYMVm/FPR2 on M1 microglia in vitro, we further explored the effect of WKYMVm on microglia in the spinal cord after injury in rats. As shown in Figure 7(a), iNOS was increased significantly in the SCI group, as shown by immunohistochemistry, and WKYMVm treatment reversed this effect. In addition, iNOS protein expression was examined in damaged segment samples, and the results showed that SCI significantly increased iNOS expression, and this effect was inhibited by WKYMVm (Figure 7(b)). Furthermore, immunofluorescence costaining of iNOS and Iba-1 was performed, and the numbers of iNOS- and Iba-1-positive cells were elevated in the SCI group, while WKYMVm reversed the level of iNOS in the SCI group (Figure 7(c)). These data showed that WKYMVm/FPR2 blocked microglial polarization to the M1 subtype in the injured spinal cord.

### 3.8. WKYMVm Improved the Inflammatory Microenvironment after SCI

To investigate whether FPR2 activation by WKYMVm could inhibit the levels of proinflammatory cytokines in vivo, the production of TNF-*α*, IL-6, and IL-1*β* was measured in the samples of damaged sections at 3 days after SCI by RT-PCR and ELISA. As shown in Figures 7(d)–7(f), the mRNA levels of TNF-*α*, IL-6, and IL-1*β* were increased after SCI, whereas the mRNA levels of these three cytokines were inhibited by WKYMVm. The secretion of TNF-*α*, IL-6, and IL-1*β* in the SCI+WKYMVm group was surely lower than that in the SCI group, as determined by ELISA (Figures 7(g)–7(i)). Our results showed that WKYMVm inhibited the production of proinflammatory cytokines such as TNF-*α*, IL-6, and IL-1*β* in the impaired spinal cord.

### 3.9. WKYMVm Reduced Tissue Damage and Functional Decline after SCI

**Figure 8:**
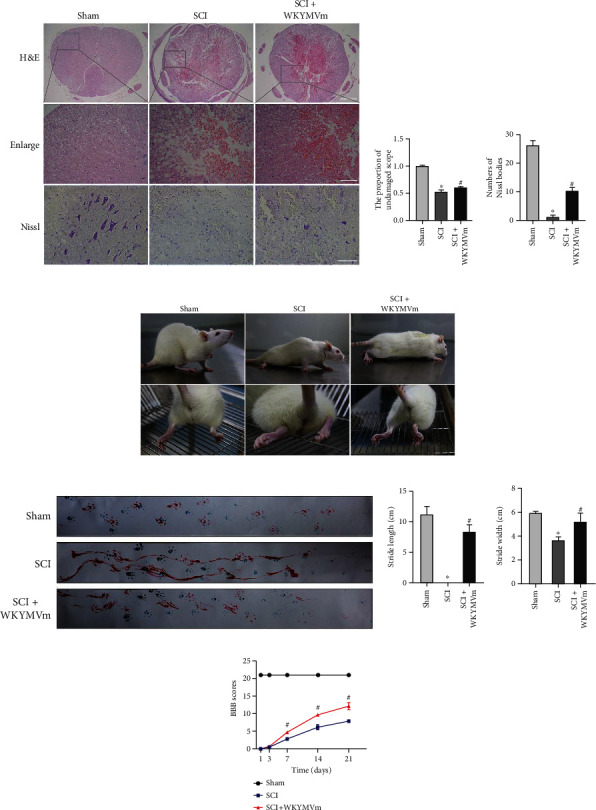
WKYMVm reduced tissue damage and functional decline after SCI. (a) Representative images from H&E and Nissl staining at 21 d after surgery. Quantitative data for (b) H&E and (c) Nissl staining. (d) Images displaying weight-bearing walking and hind limbs movements for the sham, SCI, and SCI+WKYMVm groups. Red arrows indicate weight-supported stepping. (e) The footprint analysis of rats from different groups. (f) Quantification of rats' stride length and stride width in footprint analysis. (g) Basso, Beattie, and Bresnahan (BBB) scores after surgery. Scale bars: 500 *μ*m (H&E); 200 *μ*m (enlarge); 100 *μ*m (Nissl). ^∗^*p* < 0.05 vs. the sham group; ^#^*p* < 0.05 vs. the SCI group.

To investigate the neuroprotective effect of WKYMVm/FPR2, paraffin sections were stained with H&E and Nissl. The results showed that damage to the central gray matter and peripheral white matter in the SCI group was more significant than that in the sham group, and the structural disorder of the peripheral white matter was more serious in the SCI group. However, tissue destruction in the SCI+WKYMVm group was less than that in the SCI group (Figures 8(a) and 8(b)). Furthermore, paraffin sections were stained with Nissl, and the number of Nissl-positive foci was counted. The results showed severe neuronal loss in the SCI group, while the amount of Nissl staining in the spinal cord central gray matter was higher in the SCI+WKYMVm group than in the SCI group (Figures 8(a) and 8(c)). These data suggested that WKYMVm has protective effects on damaged neurons and spinal cord tissue after SCI.

Weight-bearing walking and footprint analysis were evaluated at 21 days after the operation, which directly reflected motor function. As shown in Figure 8(d), rats in the sham group could easily support their weight on both hind limbs. However, SCI group rats could barely support their weight on their hind limbs. Notably, rats in the SCI+WKYMVm group were able to support their body weight with one hind limb or occasionally both hind limbs. Footprint analysis showed coordination differences in the hind limbs. As shown in Figures 8(e) and 8(f), the footprints of rats in the sham group were clear without dragging. In stark contrast to those in the sham group, untreated SCI rats showed significant dragging of their hind limbs (red footprints). As expected, WKYMVm-treated SCI rats exhibited fairly consistent hind limb trajectories at 21 days after injury, with little dragging. In addition, we quantified motor function by determining BBB scores. The BBB scores were measured at 1, 3, 7, 14, and 21 days after contusion to evaluate whether WKYMVm could protect against motor function decline. At 1 and 3 days after contusion, the BBB scores in the SCI group and SCI+WKYMVm group showed no significant differences (Figure 8(g)). However, BBB scores were higher in the SCI+WKYMVm group than those in the SCI group after 7 days (Figure 8(g)). These results suggested that WKYMVm could reduce tissue damage and dysfunction after SCI.

## 4. Discussion

**Figure 9:**
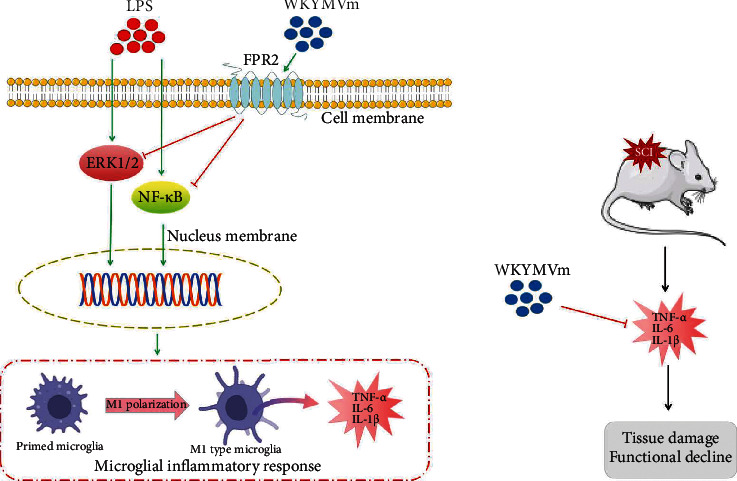
Working model showing the effect of WKYMVm in vitro and in vivo. (a) WKYMVm suppresses the ERK1/2 and NF-*κ*B signaling pathways, attenuates M1 microglial polarization, and reduces inflammatory cytokines expression in vitro. (b) WKYMVm reduces tissue damage and functional decline after SCI via inhibiting inflammatory cytokines in vivo. LPS: lipopolysaccharide; FPR2: formyl peptide receptor 2; ERK1/2: extracellular response kinases 1 and 2; NF-*κ*B: nuclear factor-*κ*B; SCI: spinal cord injury.

In this study, we demonstrated that FPR2 activation by WKYMVm exerted obvious inhibitory effects by blocking the release of proinflammatory cytokines in LPS-stimulated microglial cells (including HAPI microglia and primary microglia) and SCI rats. This study provided the following evidence: (1) WKYMVm promotes the expression of FPR2 in microglial cells; (2) FPR2 activation by WKYMVm attenuates ERK1/2 and NF-*κ*B signaling in LPS-stimulated microglial cells; (3) FPR2 activation by WKYMVm reduces the secretion of proinflammatory mediators and M1 polarization in microglial cells; (4) treatment with WKYMVm inhibits M1 microglial polarization and the production of proinflammatory mediators in damaged spinal cord; and (5) FPR2 activation by WKYMVm alleviates secondary injury after SCI and reduces tissue damage in rats. To the best of our knowledge, this is the first study confirming the role of FPR2 and WKYMVm in the regulation of the inflammatory response after SCI and describing the suppression of the inflammatory response by WKYMVm-activated FPR2 through negative modulation of the ERK1/2 and NF-*κ*B p65 signaling pathways in microglia (Figure 9).

SCI is a clinical problem associated with high mortality and disability rates [45]. The pathological process of SCI is divided into two stages: primary and secondary injury. Primary injury is caused by direct mechanical force. Secondary injury is secondary to mechanical force and involves a complex array of molecular reactions, including disruption of the ionic steady state, local edema, ischemia, focal hemorrhage, oxidative stress, and inflammatory responses [46]. The acute inflammatory process is an innate host defense mechanism against invading pathogens and tissue damage. Excessive secretion of proinflammatory mediators and neurotoxic molecules is one of the important reasons for the inhibition of functional recovery and aggravation of neuropathic paresthesia in the damaged spinal cord [47, 48]. Modulating inflammatory resolution has been proposed as a novel strategy to treat a variety of CNS diseases. Therefore, reducing the inflammatory response during secondary injury may be a potential strategy for SCI therapy. In this study, we explored the relationship between the secretion of inflammatory factors and the inflammatory environment of SCI in vivo and in vitro. We found that the production of various inflammatory factors, such as TNF-*α*, IL-6, and IL-1*β*, was significantly elevated in LPS-stimulated microglial models and a rat model of SCI. Our findings were consistent with those of previous studies [27, 28].

FPRs form high-order structures (e.g., FPR1/FPR2 heterodimers, FPR2 homologous dimers, and FPR1 homologous dimers) that cause changes in downstream intracellular signaling pathways, allowing the colocalization of effector domain, enhancing intracellular activation, or creating new ligand specificity [49, 50]. Some studies have shown evidence that the function of FPRs is associated with many diseases, such as inflammatory disorders, cancer, and infections [51, 52]. Recently, many studies have shown that the anti-inflammatory effects of FPRs are mainly induced by the FPR2 receptor. FPR2 was initially identified in leukocytes and was later found to be widely expressed in phagocytic cell types, such as neutrophils, macrophages, dendritic cells, and stem cells [53, 54]. FPR2 is also richly expressed in microglial cells [55–57]. Consistent with this conclusion, our results showed that FPR2 was expressed in HAPI microglia and primary microglia and increased with increasing concentrations of the agonist WKYMVm. FPR2 can exert both anti-inflammatory and proinflammatory effects, and this conflicting function is determined by the properties of the ligands and the formation of higher order structures. Annexin-1 and Lipoxin A4 are ligands that induce the anti-inflammatory effects of FPR2 [56, 57]. Recently, it has been reported that the synthetic FPR2 agonist WKYMVm has a strong anti-inflammatory effect and can inhibit the secretion of proinflammatory cytokines and promote the expression of anti-inflammatory cytokines [34].

WKYMVm, which is an immunomodulatory peptide with a strong affinity for FPR2, has recently been reported to have multiple effects on cell differentiation [31] and can also stimulate chemotaxis in a variety of cells, such as monocytes, neutrophils, and B lymphocytes [58, 59]. Previous studies have shown that WKYMVm inhibits the levels of key inflammation- and immune-related genes in multiple injury or disease models and exerts prominent anti-inflammatory effects on tissues such as the bone and lung [31, 60]. In addition, Hu and colleagues demonstrated that the WKYMVm peptide suppressed osteoclastogenesis by downregulating the levels of proinflammatory factors such as TNF-*α* and IL-1*β* [36]. Other FPR2 agonists, such as Annexin-1 and Lipoxin A4, have previously been reported to promote recovery from SCI [56, 57]. However, the effects of WKYMVm on microglial cells and SCI have rarely been reported. Accordingly, we have hypothesized that WKYMVm suppressed SCI under inflammatory conditions. Our results demonstrated that WKYMVm reduced LPS-induced microglial inflammation, which is consistent with previous reports that WKYMVm suppresses the inflammatory response in BMMs, RAW 264.7 cells [36], and HUVECs [61]. In this study, we showed that WKYMVm negatively regulated inflammation in vitro. In addition, WKYMVm also reduced proinflammatory factors in damaged tissues after SCI. Consistent with this observation, the LPS-stimulated polarization of M1 microglia was partially reversed by WKYMVm, the degree of amoeba-like changes was reduced, and the level of classical M1 microglial markers, such as iNOS, was decreased. Our cell morphology results are consistent with those of previous reports [62]. Therefore, we believe that the inhibition of inflammatory factor expression after FPR2 activation by WKYMVm may be achieved by inhibiting M1 microglial polarization.

Tylek and colleagues examined the beneficial antioxidant and resolving effects of FPR2 agonists on LPS-stimulated microglia and found that p38, ERK1/2, and NF-*κ*B may be crucial signaling pathways [63]. Furthermore, it has been reported in the literature [63, 64] that the NF-*κ*B and MAPK pathways are important therapeutic targets for controlling inflammation in the CNS. Thus, blocking these signaling pathways may have protective effects against SCI. Based on these research results, we explored the potential mechanism by which WKYMVm/FPR2 inhibits the activation of the proinflammatory microglial subtype in vitro. First, we investigated the effect of WKYMVm/FPR2 on the p38 and ERK1/2 signaling pathways. The results showed that FPR2 activation by WKYMVm could reduce the phosphorylation of ERK1/2 but not p38. Our observations are not entirely consistent with those of other reports that FPR2 stimulation induced the phosphorylation of multiple signaling proteins, including p38MAPK and ERK1/2 [65, 66]. The difference may be related to the nature of the agonists that FPR2 acts on, and different agonists have different effects on MAPK signaling pathways [63]. Furthermore, we examined the relationship between FPR2 activation by WKYMVm and the NF-*κ*B p65 signaling pathway. Our results demonstrated that WKYMVm/FPR2 upregulated the level of I*κ*B*α* and downregulated the level of p-p65, thereby blocking activation of the NF-*κ*B signaling pathway, which is consistent with previous findings of other agonists [36, 63]. These findings indicated that WKYMVm/FPR2 inhibits the NF-*κ*B signaling pathway in microglial cells under inflammatory conditions. However, the specific effect of WKYMVm/FPR2 on the MAPK signaling pathway is not clear, and the research conclusions are inconsistent and require further research.

Another important finding in this study is that WKYMVm/FPR2 effectively inhibited the inflammatory response in rats. Our results showed that intraperitoneal injection of WKYMVm reduced iNOS production at the site of SCI. In addition, WKYMVm significantly attenuated the production of TNF-*α*, IL-6, and IL-1*β* in the damaged tissue of rats, which was consistent with our in vitro results. These results suggested that the activation of FPR2 by WKYMVm alleviated the inflammatory response in the damaged spinal cord, which may be achieved by inhibiting M1 microglial polarization. In addition, we found that WKYMVm/FPR2 reduced structural destruction, neuronal loss, and functional decline in SCI rats, suggesting that WKYMVm is an effective molecule for alleviating traumatic SCI.

Our study has several limitations. First, due to time constraints, we did not knock down the FPR2 receptor to observe whether WKYMVM has any effect on microglial cells when FPR2 is low expressed. Second, we only performed simple signal pathway correlation verification without further exploration. Finally, whether WKYMVm/FPR2 can reduce tissue damage through mechanisms other than inhibiting inflammation remains to be studied.

In summary, our results provided evidence for the role of WKYMVm/FPR2 in the inflammation regulation that microglial cells are involved. FPR2 activation by WKYMVm inhibits microglial M1 polarization and inflammatory factor secretion and reduces tissue destruction and functional decline in SCI by modulating the ERK1/2 and NF-*κ*B signaling pathways. Moreover, our results indicated that FPR2 activation by WKYMVm may have potential developmental value in the treatment of SCI.

## Figures and Tables

**Table 1:** PCR primers in this study.

Gene	Sequence	Accession
FPR2	F 5′-GAGCCTGGCTAGGAAGGTG-3′	NM_008039.2
	R 5′-TGCTGAAACCAATAAGGAACCTG-3′	
iNOS	F 5′-GTTCTCAGCCCAACAATACAAGA-3′	NM_010927.4
	R 5′-GTGGACGGGTCGATGTCAC-3′	
TNF-*α*	F 5′-ATGGGCTCCCTCTCATCAGT-3′	NM_013693.3
	R GCTTGGTGGTTTGCTACGAC-3′	
IL-6	F 5′-AACCACGGCCTTCCCTACTTCA-3′	NM_001314054.1
	R 5′-TCATTTCCACGATTTCCCAGAG-3′	
IL-1*β*	F 5′-AGGAGAGACAAGCAACGACA-3′	NM_008361.4
	R 5′-CTTTTCCATCTTCTTCTTTGGGTAT-3′	
GAPDH	F 5′-CATCACTGCCACCCAGAAGACTG-3′	NM_036165840.1
	R 5′-ATGCCAGTGAGCTTCCCGTTCAG-3′	

## Data Availability

All data are fully available without restriction.

## Abbreviations

SCI: Spinal cord injury
CNS: Central nervous system
MAPKs: Mitogen-activated protein kinases
ERK1/2: Extracellular response kinases 1 and 2
NF-*κ*B: Nuclear factor-*κ*B
FPRs: Formyl peptide receptor
FPR2: Formyl peptide receptor 2
TNF-*α*: Tumor necrosis factor-*α*
IL-6: Interleukin-6
IL-1*β*: Interleukin-1*β*
iNOS: Inducible nitric oxide synthase
Iba-1: Microglia marker protein
GAPDH: Glyceraldehyde 3-phosphate dehydrogenase
PBS: Phosphate-buffered saline
DAPI: 4′-6-Diamidino-2-phenylindole
ELISA: Enzyme-linked immunosorbent assay
RT-PCR: Real-time polymerase chain reaction
PVDF: Polyvinylidene difluoride
ECL: Enhanced chemiluminescence
DMEM: Dulbecco's modified Eagle medium
FBS: Fetal bovine serum
BCA: Bicinchoninic acid disodium
TBST: Tris-HCl-based buffer with Tween 20
FPA: Paraformaldehyde
H&E: Haematoxylin and eosin
HAPI: Highly aggressive proliferating immortalized
IHC: Immunohistochemical
LPS: Lipopolysaccharide
mRNA: Messenger RNA
BBB: Basso, Beattie, and Bresnahan
